# Osteoimmunology: The Regulatory Roles of T Lymphocytes in Osteoporosis

**DOI:** 10.3389/fendo.2020.00465

**Published:** 2020-08-11

**Authors:** Wenjuan Zhang, Kai Dang, Ying Huai, Airong Qian

**Affiliations:** Lab for Bone Metabolism, Xi'an Key Laboratory of Special Medicine and Health Engineering, Key Lab for Space Biosciences and Biotechnology, Research Center for Special Medicine and Health Systems Engineering, NPU-UAB Joint Laboratory for Bone Metabolism, School of Life Sciences, Northwestern Polytechnical University, Xi'an, China

**Keywords:** osteoimmunology, T lymphocytes, osteoporosis, bone formation, bone resorption

## Abstract

Immune imbalance caused bone loss. Osteoimmunology is emerging as a new interdisciplinary field to explore the shared molecules and interactions between the skeletal and immune systems. In particular, T lymphocytes (T cells) play pivotal roles in the regulation of bone health. However, the roles and mechanisms of T cells in the treatment of osteoporosis are not fully understood. The present review aims to summarize the essential regulatory roles of T cells in the pathophysiology of various cases of osteoporosis and the development of T cell therapy for osteoporosis from osteoimmunology perspective. As T cell-mediated immunomodulation inhibition reduced bone loss, there is an increasing interest in T cell therapy in an attempt to treat osteoporosis. In summary, the T cell therapy may be further pursued as an immunomodulatory strategy for the treatment of osteoporosis, which can provide a novel perspective for drug development in the future.

## Introductions

Osteoporosis is a prevailing metabolic bone disease in both men > 50 years and postmenopausal women, which increases bone fragility and may further result in bone fractures, thus significantly leading to serious health problems for patients (1). Worldwide, nearly 200 million people are diagnosed with osteoporosis annually, even leading to almost 9 million osteoporotic fractures (2). In the US, it was approximately 53.6 million of the adult population of years > 50 who suffered from osteoporosis and low bone mass (54% of the population) (3). In fact, osteoporosis patients not only suffer from the enormous pain and disability but also bring a huge economic burden for patients and their families. In the US, it has been estimated that the financial costs associated with bone fractures will reach $25.3 billion by the end of 2025 (4).

In traditional view, osteoporosis was considered as the imbalance of bone remodeling between osteoclasts and osteoblasts (5). Recently, the immune system was reported to regulate the bone system, which promoted the emergence of interdisciplinary field of osteoimmunology (6–9). The immune and bone systems share the same microenvironment. The immune system regulates osteocytes by the secretion of inflammatory factors and related ligand, which further affects bone formation and bone resorption (8, 10). T cells, B cells, and cytokines are important regulatory factors in the bone resorption. Among them, T cells play pivotal roles in the regulation of bone remodeling (11, 12). The osteoclast differentiation was enhanced, and the bone mineral density was decreased in the nude mice (T cell deficient), which was due to the immune imbalance of T cells promoting osteoclast differentiation and bone resorption (13, 14). In pathophysiological condition, activated T cells secreted multiple inflammatory factors and related ligands such as TNF-α, IL-1, IL-6, IL-17, and CD40L, which enhanced bone resorption and disrupted bone balance, resulting in bone loss (15, 16). Th17 cells are mainly involved in inducing bone resorption (osteoclastogenesis) (17), while Treg cells are major suppressors of bone loss (18, 19) by inhibiting differentiation of monocytes into osteoclasts (17, 20, 21). These reports indicated that immune imbalance promoted osteoclast differentiation, further leading to bone loss. However, the roles of T cells in osteoporosis and the underlying mechanism of T cells in the regulation of bone system are still unclear.

Recently, there is an increasing interest in immune therapies especially T cell therapies for the treatment of osteoporosis (22). For example, antiretroviral therapy worsens HIV-induced bone loss (23), which may be an important future approach to treat osteoporosis in human. That is because T cell reconstitution induces RANKL and TNFα production by B-cells and/or T-cells, which further enhancing bone resorption and bone loss. T cell therapy became the effective strategy for the treatment of osteoporosis. For example, RANKL/RANK inhibition may be an attractive approach for the treatment of postmenopausal osteoporosis (24). Sclareol is a natural product (initially isolated from the leaves and flowers of *Salvia sclarea*) with immune regulation and anti-inflammatory effects, and it prevents ovariectomy-induced bone loss *in vivo* and inhibits osteoclastogenesis *in vitro* via suppressing NF-κB and MAPK/ERK signaling pathways (25). Thus, it will be essential to develop T cell therapy that may be a huge potential for the treatment of osteoporosis in future clinical applications.

Herein, we briefly highlight the roles of T cells in various types of osteoporosis and uncover novel mechanisms of osteoimmunology, which provides new insight for clinical implications in the treatment of osteoporosis. Nonetheless, the underlying mechanisms of bone-immune interactions need to be further dissected, and an accumulative evidence continues to be made in favor of regulation roles of immune cells in osteoporosis. Most importantly, the T cell therapy may represent a suitable and potential approach to reinstate aberrant bone remodeling in the bone metabolism diseases.

## Osteoimmunology and The Regulation of T Cell Cytokines in Osteoporosis

Osteoimmunology is the intricate interaction between the immune system and the bone system (6–9). The RANKL/RANK/OPG pathway is essential for the differentiation of bone-resorbing osteoclasts and immune regulation (26, 27). Activated T cells directly produce RANKL, which further stimulates osteoclast formation (28, 29). RANKL and RANK were identified as key factors in the mediation of bone remodeling, especially in the osteoclast formation (29, 30). Furthermore, the activated RANK facilitated the expression of tumor necrosis factor (TNF) receptor-associated factors (TRAFs), such as TRAF6, which leads to osteoclast differentiation (31, 32). In OVX mice, the low-dose RANKL of CD8^+^ Treg cells decreased the expression of inflammatory and osteoclastogenic cytokines, thus suppressing bone resorption (33). Multiple cytokines produced by T cell including interleukin (IL)-12, IL-17, IL-18, and TNF-α were involved in RANK signaling, and thus play essential roles in regulating osteoclastogenesis and osteoclast differentiation (34). In addition, activated T cells suppress osteoclast differentiation by the antiviral cytokine IFN-γ (35). Various inflammatory cytokines were necessary and sufficient for bone metabolism (11). IL-17A also upregulates the expression of RANK, thus promoting the osteoclastogenic activity of RANKL (36). All these studies indicated that T cell cytokines play essential roles in osteoporosis, which may be the potential targets for the treatment of osteoporosis. Various T cell cytokines are listed in Table 1.

**Table 1:** Roles of various T cell cytokines in the regulation of osteoclastogenesis.

**Cytokine**	**Source**	**Modulation of immunology**	**Osteoclastogenic function**	**References**
RANKL	Th17 cells	Osteoclast differentiation dendritic cells (DCs) maturation	Osteoclast activation via RANK	(37)
RANK	Osteoclasts, DCs	DCs activation	Osteoclast differentiation and activation	(38)
OPG	Osteoclasts	Decoy receptor for RANKL	Inhibits osteoclastogenesis	(39)
TNFα	Th17, macrophage DCs	Pro-inflammatory cytokine	Indirect osteoclastic activation through RANKL	(37)
M-CSF	Th1	Pro-inflammatory	Inhibits osteoclastogenesis	(38)
IL-4	Th2	Humoral immunity	Inhibits osteoclastogenesis	(40)
IL-6	Macrophage, DCs	Pro-inflammation, Th17 induction	Activation of osteoclastogenesis	(41)
IL-7	T cells	Pro-inflammatory cytokine	Inhibits osteoclast formation	(42)
IL-8				
IL-10	Regulatory T (Treg)	Anti-inflammatory	Suppress bone resorption	(43)
IL-17	T cells	Pro-inflammatory cytokine	RANKL expression and vigorous pro-inflammatory potency	(44)
IL-27	Macrophage and DCs	Th1and Treg Th17 induction	Inhibits osteoclast formation, blocking receptor activator of NF-κB (RANK)-dependent osteoclastogenesis	(45)
IL-12	Antigen-presenting cells	Pro-inflammatory cytokine	Inhibits RANKL-stimulated Osteoclastogenesis	(46)
IL-15	NK cells	Pro-inflammatory cytokine	Enhances RANK ligand (RANKL) and macrophage colony-stimulating factor expression	(47)
IL-23	Macrophage and DCs	Th17 induction	Indirect osteoclast activation	(48)
IFN-γ	Th1, NK cells	Cellular immunity	Inhibits osteoclastogenesis	(41)

## The T Cells in The Regulation of Various Osteoporosis

T cells perform a dual role in the regulation of bone remodeling: resting T cells protect osteoclasts from bone resorption, and activated T cells actively regulate the osteoclasts generation. This review aims to summarize the regulatory roles of T cells in various types of osteoporosis such as chronic inflammation-induced osteoporosis, senile osteoporosis, estrogen deficiency-induced osteoporosis, parathyroid hormone (PTH)-induced osteoporosis, and glucocorticoid-induced osteoporosis (GIO).

### The Regulatory Roles of T Cells in Chronic Inflammation-Induced Osteoporosis

Osteoporosis commonly occurred in various chronic inflammatory diseases, such as rheumatic arthritis (RA), gout, psoriatic disease, osteoarthritis, and axial spondylarthritis and even leads to functional disability and increased mortality (49–52). It is interesting to note that Tregs play pivotal roles in inflammation-induced bone loss by inhibiting the functions of Th17 cells (19, 53). In particular, Foxp3+ Treg cells play an indispensable role in bone and hematopoietic homeostasis acting on osteoclast development and function (54). In addition, in inflammation condition, the expression of nuclear factor of activated T cells cytoplasmic 1 (NFATc1), as well as by inflammatory cytokines such as TNFα, IL-1β, and IL-6 was induced and produced to promote osteoclast differentiation mediated by the RANKL-RANK and calcium signaling (8). INFγ, the main Th1 cytokine, can strongly inhibit osteoclast differentiation *in vitro* through the proteasomal degradation of TRAF6, indicating that T cells regulate osteoclastogenesis (28). The T cell subset, Tregs, also suppresses osteoclast formation and bone resorbing *in vitro* (53). CTLA-4 is the most essential regulator in the Treg-mediated inhibition of osteoclast differentiation, whereas the major cytokines of Tregs-TGFβ and IL-10 do not possess any essential roles (53). All these studies suggest that T cells and their related cytokine play pivotal roles in the regulation of osteoporosis, and they may be the potential therapeutic targets for bone loss.

Generally, chronic inflammatory diseases are associated with bone resorption. HIV-infected men had low CD4 T cells, which is inversely associated with bone loss (55). Some studies suggest that T cells are not associated with bone mineral density in HIV-infected patients treated with combination antiretroviral therapy (cART) (56). However, cART seems to influence bone mineral density (BMD) with the protective effect. Therefore, the regulatory roles for activated T cells in the pathogenesis of osteoporosis warrant further investigation. In RA patients, the enhanced osteoclast differentiation and activation lead to bone erosion and systematic osteoporosis (57). Indeed, inflammatory cytokines including RANKL, TNFα, IL-6, and IL-1 were elevated in RA patients, which promoted the osteoclast differentiation (58). Taken together, these studies suggest that the T cells may determine the osteoclast differentiation in the chronic inflammatory diseases, and the T cell regulatory therapy could potentially have significant impact on the drug development for osteoporosis. However, whether the T cell therapy is efficient for osteoporosis in clinical studies needs further investigation.

### The Regulation Roles of T Cells in Senile Osteoporosis

Aging is always accompanied with the imbalance between bone formation and resorption, causing skeletal microarchitecture damage and bone loss (59). The production of naïve T cells is severely impaired due to a decreased output of lymphoid cells from the bone marrow and the deterioration of the thymus (60). Incidence and severity of osteoporosis are increased in the older population (61). The prevalence of low BMD is associated with immune activation and senescence induced by HIV infection (62). Total T cells were increased in the bone marrow (BM) with age, especially the highly differentiated CD8^+^ T cells without the expression of the co-stimulatory molecule CD28, while natural killer T (NKT) cells, monocytes, and naïve CD8^+^ T cells were decreased in the BM with age (63). It seems that the immune system abnormality plays important roles in the regulation of senile osteoporosis.

Recent discoveries suggest that T cell dysfunction induced the accumulation of cytokines, immunological mediators, and transcription factors, which affect osteoclast and osteoblast in the elderly (64). Cytokines such as IL-6, TNF-α, and IL-1 increased with age (65, 66). IL-1 and TNF-α activate the inducible NOS (iNOS) pathway, which inhibited osteoblast differentiation and enhanced osteoblast apoptosis *in vitro* (67). IL-12 derived from T cells, alone or combined with IL-18, was identified to inhibit osteoclast formation *in vitro* (68). IL-4 regulated osteoclast differentiation through the antagonism between STAT6 and NF-kB signaling (69). In addition, T cell mediated the bone balance by the inhibition of osteoclastogenesis through the crucial immunoregulatory control, mainly OPG expression and simultaneous production of cytokines (64). IFN-g, IL-12, and IL-18 inhibited the RANKL-induced maturation and activation of osteoclasts (64). Furthermore, senescent T cells impaired the production of IFN-γ, OPG, and osteoclast-inhibiting cytokines, which increased the incidence of aged osteoporosis. In addition, cytokines such as TGFβ and RANKL secreted by activated T cells can activate p38 MAPKs and further regulate bone development and remodeling. P38α MAPK mediates osteoclast proliferation and bone remodeling in an aging-dependent manner (70). Overall, T cells and their cytokines play important roles in the regulation of aged osteoporosis, which may be the novel targets for the treatment of osteoporosis, suggesting that T cell therapy could be used as immunotherapy and may be beneficial in counteracting immunosenescence in old population. Meanwhile, in females, osteoporosis occurrence is generally attributed to the decrease in estrogen, thus leading to estrogen deficiency-induced osteoporosis. The underlying mechanisms of T cells involved in the mediation of the postmenopausal osteoporosis were dissected in the next section, The Regulatory Roles of T Cells in Estrogen Deficiency-Induced Osteoporosis.

### The Regulatory Roles of T Cells in Estrogen Deficiency-Induced Osteoporosis

The loss of estrogen initiates the inflammatory changes of bone-microenvironment state, inducing a rapid phase of bone loss leading to osteoporosis in half of postmenopausal women. In postmenopausal women, estrogen deficiency stimulates CD4^+^ T cell dysregulation and induces elevated circulating levels of inflammatory cytokines, especially TNFα, IFN-γ, IL-17, RANKL, and CD40L (71–74). These cytokines exert impressive regulatory effects on bone resorption. For example, TNF-α was overexpressed in the BM in postmenopausal osteoporosis, which promotes RANKL-induced osteoclast formation through the activation of NF-κB and PI3K/Akt signaling (74). Besides, TNF-α was identified to induce both autophagy and apoptosis in osteoblasts to enhance bone loss in postmenopausal women (75). Besides, estrogen deficiency increased the number of the costimulatory factors, CD40L, expressed on activated T cells, inducing the expressions of M-CSF and RANKL on stromal cells and downregulating the production of OPG, ultimately resulting in a remarkable increase in osteoclast numbers (76, 77). The pro-osteoclastic cytokines, such as IL-6, TNF-α, and IL-1, were increased significantly in estrogen deficiency-induced osteoporosis (78). All these studies indicated that the inflammatory cytokines and costimulatory factors of T cells changed significantly in estrogen deficiency-induced osteoporosis, which may provide the novel perspective for the treatment of bone loss in postmenopausal women.

Moreover, estrogen deficiency stimulates the IL-17 differentiation of Th17 cells (79) and augments the expression levels of pro-osteoclastogenic cytokines, such as TNF-a, IL-6, and RANKL, ultimately leading to bone loss. Nevertheless, IL-17 receptor deficiency induced more serious bone loss in OVX mice than that in control groups, implying that IL-17 may possess the bone protective effects (80). The pro-osteoclastogenic cytokine changes were reversed with the supplementary oral estrogen, indicating that estrogen may suppress Th17 differentiation and IL-17 production to protect bone health (81). In summary, in postmenopausal women, both aging and hormonal deficiency stimulate the deregulation of T cells contributing to the inflammatory, which increased bone resorption, resulting in a bone loss or osteoporosis. We believe that focusing on the potential biological mechanisms of T cells is of paramount importance for developing novel therapy strategies for the treatment of postmenopausal osteoporosis. However, further confirmation in phase I/II trials is needed to validate these strategies in a broader clinical evaluation.

### The Regulatory Roles of T Cells in PTH-Induced Osteoporosis

PTH is a key calciotropic hormone and a critical regulator for postnatal skeletal development (82). The secretion of inflammatory or osteoclastogenic cytokines of T cells and bone cells was facilitated under long-term PTH administration, such as RANKL, TNF-α, and IL-17, which promoted the bone resorption (83). PTH induced bone loss via the expansion of intestinal TNF^+^ T and Th17 cells, and the increase in their S1P-receptor-1 mediated egress from the intestine and recruitment to the BM (84). So targeting the gut microbiota or T cell migration may represent novel therapeutic strategies for PTH-induced osteoporosis. In addition, PTH exploited CD4^+^ T cells to induce TNFα production that enhances the formation of IL-17A secreting Th17 T cells. Both TNFα and IL-17 further facilitated the development of an increased RANKL/OPG ratio favorable to osteoclastic bone resorption (85). Moreover, PTH boosted the production of TNF-α and RANKL in CD4^+^ T cells, which triggered osteoclastogenic generation and bone resorption activity (86). Clinical studies also showed that PTH treatment increased Th17 cell numbers and the IL-17 production in humans with primary hyperparathyroidism (34). IL-17 intensified PTH-induced bone loss through the stimulation of the RANKL production in osteoblast-lineage cells, which is parallel to the roles of IL-17 in estrogen deficiency-induced osteoporosis.

Notably, T cells also secreted PTH receptors involved in the regulation of trabecular bone development (87). For example, T cells promoted the signals of BMSC proliferation through the combination of CD40L on T cells and its receptor on BMSC, weakening the bone catabolic activity of cPTH, leading to a reduction of the RANKL to OPG ratio and osteoclastogenic activity (88). Several studies found that the intermittent PTH administration at low dosage increased bone formation and bone mass, thus attenuating bone loss (89, 90). The deletion of PTH receptor in BM mesenchymal progenitors results in a rapid increase in BM adipocyte accompanied with the reduction of bone mass. Given the essential regulatory roles of T cells for the PTH-induced bone loss, particular attention will be paid toward the combinations of intermittent PTH (iPTH) and T cell therapy for PTH-induced osteoporosis.

### The Regulatory Roles of T Cells in GIO

Glucocorticoids (GCs) are extensively used for the treatment of immune and inflammatory disorders due to their powerful immunosuppressive and anti-inflammatory actions (91, 92). However, long-term exogenous GC therapy might cause rapid and pronounced bone loss and subsequently osteoporosis (93, 94). The pathogenesis of GIO was predominantly attributed to the fact that GCs impaired bone formation by reduction of osteoblast differentiation and activity via the expression of the osteoblast-specific transcription factor runt-related Runx2 (95–97). In addition, the long-term GC administration affects bone remodeling by whittling the insulin-like growth factor (IGF) in ossification (98). GCs enhanced the expression levels of RANKL in both osteoblasts and stromal cells, which triggered osteoclastogenesis and activated osteoclastic bone resorption by binding to the RANKL receptor RANK (99), thus resulting in the primary phase of rapid bone loss. On the other hand, GCs contributed to the apoptosis of certain T cell subsets, further augmented the secretion of RANKL, and directly induced osteoclast differentiation (100). Interestingly, different T cell subsets exhibit distinct sensitivity to GC-induced apoptosis. For example, Th17 cells, as an osteoclastogenesis-promoting factor, are resistant to GC-induced apoptosis and cytokine suppression mostly through the high production of IL-17 and RANKL (79). Therefore, GC therapy fails to inhibit the Th17 cell activation and the IL-17 and RANKL production. Excessive GCs could reduce the production of OPG, further promoting osteoclast differentiation and resulting in bone resorption. Given above, we assert that T cell therapy may be effective for the GC-induced osteoporosis.

## T Cell Therapy for Osteoporosis

T cells and their secreted cytokines are responsible for bone resorption in various osteoporosis. T cell therapy may be a potentially therapeutic approach to osteoporosis. For example, anti-inflammatory therapies have shown good potential in an animal model, although they have not been widely used clinically to treat osteoporosis (101). Immune modulation therapy such as probiotics was considered as a novel strategy for bone loss (102–104). RANKL was considered as an activator of dendritic cell (DC) expression in T cells. Anti-RANKL therapeutic antibody drug, denosumab, has been successfully applied in the treatment of osteoporosis in clinics (105–107). In addition, a novel vaccine targeting RANKL by introducing a p-nitrophenylalanine at a single site in mRANKL immunization could prevent OVX-induced bone loss in mice (108). Notably, anti-RANKL antibody inhibited osteoporosis and bone destruction, but possesses no therapeutic effect on RA disease. Therefore, it is necessary to rethink about the underlying mechanisms of bone-related diseases.

Recently, extracts and natural products derived from traditional Chinese medicine (TCM) have great potential as well as advantages in the prevention and treatment of osteoporosis in terms of good therapeutic effect, low toxicity, and side effects (109, 110), and they have gained increasing attention from the medical community. For example, polysaccharides derived from persimmon leaves down-regulated RANKL-induced activation of mitogen-activated protein kinases (MAPKs) to suppress the nuclear factor of NFATc1 expression, thus possessing anti-osteoporotic effects in OVX-induced bone loss. The natural product cyperenoic acid is a terpenoid isolated from the medicinal plant *Croton crassifolius*, and it suppressed osteoclast differentiation by inhibiting the NF-κB pathway and suppressed RANKL expression (111). Baohuoside I is an active component of Herba Epimedii with the immune regulation functions of T cells and antioxidant activity, which serves as a candidate for treating postmenopausal osteoporosis (112). All these results indicated that drugs from TCM possess anti-osteoporosis effects by the regulation of T cells, and they may show great potential as therapeutic agents for osteoporosis. However, further experimental and clinical research remains to be specifically conducted to explore the cellular and molecular mechanisms of the drugs from TCM.

## Conclusion and Perspective

The pathogen clearance of various types of osteoporosis would be impaired or would delay bone resorption due to the dysfunction of the T cells. Therefore, understanding the roles of T cells in the pathogenesis of osteoporosis and the mechanisms underlying these pathologies between the immune system and the bone system may lead to the development of new treatments for osteoporosis. However, further studies, especially clinical studies, are required to explore the safety of T cell therapy for bone loss.

## Author Contributions

WZ designed, wrote and revised the whole manuscript. YH wrote the manuscript. AQ and KD helped to revise the manuscript. All authors contributed to the article and approved the submitted version.

## Conflict of Interest

The authors declare that the research was conducted in the absence of any commercial or financial relationships that could be construed as a potential conflict of interest.

## Abbreviations

BMMs: Bone marrow macrophages
BMSCs: bone marrow stromal cells
Cbfa1: core-binding factor subunit alpha-1
DC: dendritic cell
GCs: glucocorticoids
GIO: glucocorticoid-induced osteoporosis
GSK-3β: glycogen synthase kinase 3β
IGF: insulin-like growth factor
IFN: interferon
M-CSF: macrophage-colony stimulating factor
MSCs: mesenchymal stem cells
NFATc1: nuclear factor of activated T cells cytoplasmic 1
NKT: natural killer T cells
iNOS: inducible NOS
RANKL: nuclear factor-kappa-B ligand
OVX: ovariectomized
OPG: osteoprotegerin
PTH: parathyroid hormone
T cells: T lymphocytes
TRAF6: TNF receptor associated factor 6
Runx2: Transcription Factor 2
RANK: receptor activator of NF-kB ligand
RA: rheumatoid arthritis.
